# Phycocyanin Ameliorates Colitis-Associated Colorectal Cancer by Regulating the Gut Microbiota and the IL-17 Signaling Pathway

**DOI:** 10.3390/md20040260

**Published:** 2022-04-09

**Authors:** Dongjin Pan, Bingyao Huang, Yuman Gan, Chenghai Gao, Yonghong Liu, Zhenzhou Tang

**Affiliations:** Institute of Marine Drugs, Guangxi University of Chinese Medicine, Nanning 530200, China; 15177636866@163.com (D.P.); hby03502@126.com (B.H.); gan_ym2018@163.com (Y.G.); gaoch@gxtcmu.edu.cn (C.G.)

**Keywords:** phycocyanin, colorectal cancer, microbiota, transcriptome, IL-17 pathway

## Abstract

Phycocyanin (PC) is a pigment-protein complex. It has been reported that PC exerts anti-colorectal cancer activities, although the underlying mechanism has not been fully elucidated. In the present study, azoxymethane (AOM)/dextran sulfate sodium (DSS)-induced mice were orally administrated with PC, followed by microbiota and transcriptomic analyses to investigate the effects of PC on colitis-associated cancer (CAC). Our results indicated that PC ameliorated AOM/DSS induced inflammation. PC treatment significantly reduced the number of colorectal tumors and inhibited proliferation of epithelial cell in CAC mice. Moreover, PC reduced the relative abundance of Firmicutes, Deferribacteres, Proteobacteria and Epsilonbacteraeota at phylum level. Transcriptomic analysis showed that the expression of genes involved in the intestinal barrier were altered upon PC administration, Kyoto Encyclopedia of Genes and Genomes (KEGG) pathway analysis revealed the IL-17 signaling pathway was affected by PC treatment. The study demonstrated the protective therapeutic action of PC on CAC.

## 1. Introduction

Colorectal cancer (CRC) is one of the most commonly diagnosed malignancies and a leading cause of cancer-related death worldwide, the majority of CRC cases are caused by multiple risk factors including chronic inflammation, dietary style, and specific intestinal commensals [1].

Among these risk factors, chronic inflammation is a prominent factor for CRC development. Patients who suffer from inflammatory bowel diseases (IBD), such as ulcerative colitis (UC) and Crohn’s disease (CD), have a significantly higher risk of developing colitis-associated cancer (CAC) and have a higher mortality rate compared to other CRC patients [2]. Although there are many studies supporting the relevance of CAC to intestinal inflammation, the underlying mechanism has not been fully elucidated.

The mammalian gut microbiota is highly complex and, in a dynamic equilibrium, has a profound influence on human physiology and nutrition. The gut microbiota interacting with epithelial and stromal intestinal cells prevents pathogenic infestation and regulates barrier functions, mucosal immune homeostasis, metabolism of indigestible dietary fiber, and synthesis of essential nutrients for the human body [3]. Disturbances in the composition of the gut microbiota can cause chronic inflammatory lesions and produce carcinogenic metabolites, leading to neoplasia.

In recent years, increasing evidence has shown that changes in microbial abundance and diversity are associated with the occurrence and progression of CAC. Gagniere et al. summarized studies on gut microbiota composition of general population and CRC patients, they found clear differences in gut microbiota diversity in abundance between healthy individuals and CRC patients, characterized by an increased abundance of Proteobacteria and decreased Firmicutes. As such, *Bacteroides fragilis* has been shown to be enriched in CRC patients. [4] The enterotoxigenic *B. fragilis* produces fragylisin, which lyses E-cadherin on colon cells, affects epithelial permeability and causes intestinal inflammation [5]. *Fusobacterium nucleatum* can trigger a local inflammatory response and suppress the anti-tumor immune response, which is associated with a higher risk of recurrence and shorter survival times in CRC patients [6]. Studies in CAC mouse models have also revealed significant changes in microbiota composition in chemically-induced chronic intestinal inflammation [7]. These findings suggest that the gut microbiota may be a key mediator of CAC progression. CAC is a progressive process from inflammation to cancer, so preventing or alleviating inflammation is one of the strategies for CAC intervention. Currently, drugs for IBD treatments often increase patient susceptibility to infection and even lead to adverse reactions [8]. New therapeutic strategies and drugs are urgently needed.

Phycocyanin (PC) is a pigment protein complex isolated from cyanobacteria, rhodophytes, cryptophytes and Glaucophyta [9]. In the past, PC was usually extracted from open pond cultures of cyanobacterium *Arthrospira platensis* (*Spirulina platensis*) as a result of the availability. Presently, improved productivities of photoautotrophic *A. platensis* cultures have been obtained in various enclosed photobioreactors [10]. The isolation of PC involves many steps, including cell disruption, primary isolation, purification, drying and characterization of the end products. The purity of PC is evaluated based on the ratio of A_620_/A_280_. PC preparations with A_620_/A_280_ greater than 0.7 are considered as food grade, while those with A_620_/A_280_ of 3.9 are reactive grade and greater than 4.0 are analytical grade [11]. Due to its good biocompatibility and bright color, PC has been widely used in food, pharmaceutical industries, cosmetics and other fields [10]. Numerous studies have shown that phycocyanin has a variety of biological activities, such as anti-inflammation [12], antioxidation, hypoglycemic and lipid-lowering and immune regulation [13,14,15]. PC also has excellent anti-tumor activity. Studies have shown that PC has anti-proliferative and pro-apoptotic effects on cancer cell lines in vitro, and has no obvious toxicity to normal cells [16,17]. Indeed, the role of PC in CRC cancer has been studied and PC can induce apoptosis and inhibit migration of colorectal cancer cells [17]. PC has a preventive effect on DMH-induced CRC via possible cell cycle arrest [18], down-regulation of inflammatory factors [19], up-regulation of the apoptotic proteins [20], changes in mitochondrial membrane potential, etc. [21]. PC can regulate the gut microbiota to maintain the gut barrier [22]. However, the complex interplay between gut microbiota and anti-inflammatory and antitumor activity during CAC is not fully understood.

PC may not have a specific target [23]. There is a lack of knowledge about its impact on the microflora composition of CAC mice. Hence, we employed multi-omics techniques to better understand the CAC-preventing effects of PC. In this work, we aim to disclose the role of PC in CAC using microbiota and transcriptome analyses and to explore the possible crosstalk mechanism between the gut microbiota and the gene expression profile of the colon.

## 2. Results

### 2.1. PC Inhibited Azoxymethane/Dextran Sulfate Sodium (AOM/DSS)-Induced Tumorigenesis

#### 2.1.1. PC Suppressed AOM/DSS Induced Inflammation and Body Weight Loss

**Figure 1:**
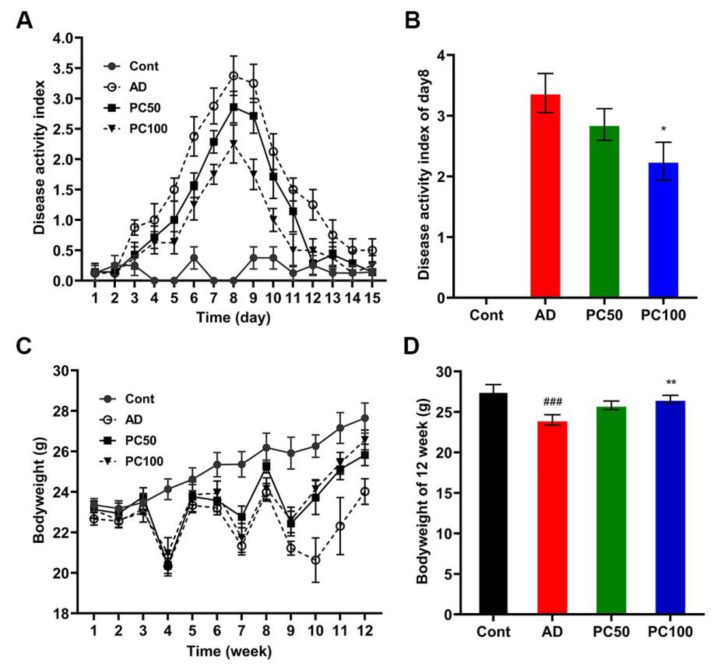
PC alleviated mice colitis symptom: (**A**) PC decreased the DAI score of AD mice; (**B**) DAI score of day eight; (**C**) Body weight change of mice during the study; (**D**) Mice body weight at 12th week. Data were expressed as mean ± SEM (*n* ≥ 8 per group). ^###^ *p* < 0.001 vs. Cont group; * *p* < 0.05; ** *p* < 0.01 vs. AD group.

We first established the CAC model by injecting mice with AOM (10 mg/kg Bodyweight) followed by three rounds of 2.5% DSS exposure. Disease activity index (DAI) analysis indicated that mice of AOM/DSS (AD) groups had higher DAI scores than the normal control (Cont) group on day eight, suggesting the induction of severe colitis (Figure 1A,B). The PC high-dose group (100 mg/kg, PC100) had a significantly lower DAI score than that of the AD group on day eight, the PC low-dose group (50 mg/kg, PC50) also had a lower DAI score compared with the AD group, although the difference was not significant (Figure 1A,B).

The average weight of mice belonging to the Cont group was higher than that of all other groups. AD mice exhibited a significant body weight loss when compared to the Cont group. PC treatment reversed the body weight loss induced by AOM/DSS (Figure 1C,D).

#### 2.1.2. PC Attenuates AOM/DSS Induced Tumorigenesis

**Figure 2:**
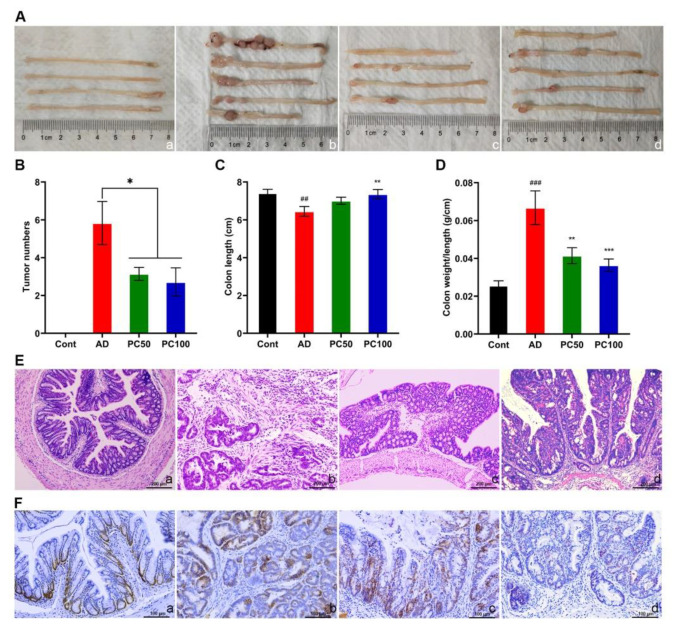
PC suppressed the tumorgenesis induced by AOM/DSS. Gross morphology of colon (**A**), tumor number (**B**), colon length (**C**) and colon weight/length ratio (**D**) were studied. (**E**) Histological observation of mice colon. Scale bars 200 µm. (**F**) PC treatment decreased the number of PCNA-positive cells in the colon. Scale bars 100 μm. a, Cont group; b, AD group; c, PC50 group; d, PC100 group. Data were presented as means ± SEM (n = 6 for AD group and *n* ≥ 7 for the other groups). ^##^ *p* < 0.01; ^###^ *p* < 0.001 vs. Cont group; * *p* < 0.05; ** *p* < 0.01; *** *p* < 0.001 vs. AD group.

After dissection, we found different numbers and sizes of tumors at the distal part of the colon of AD mice, tumor numbers were significantly reduced in PC50 and PC100 groups, yet there was no significant difference between PC50 and PC100 groups (Figure 2A,B).

The length of the mouse colon and the ratio of colon weight to length are considered to be one of the markers of the degree of intestinal inflammation [24]. The AD mice had significantly shortened colonic length (Figure 2C), and the colon weight/length ratio was significantly increased, which was markedly reversed after high dose PC treatment (Figure 2D).

The colon mucosal histological studies also revealed that damaged epithelial integrity, and abnormal glands were partially restored in PC treated groups (Figure 2E). In addition, immunohistochemical analysis showed a significant reduction in proliferating cell nuclear antigen (PCNA) positive cells in tumor tissues of mice treated with a high dose of PC (Figure 2F). These findings indicate the anti-tumorigenic effects of PC in the AOM/DSS mice.

#### 2.1.3. PC Reduces the Level of Proinflammatory Cytokines in CAC Mice

Some inflammatory cytokines such as IL-1, IL-6, IFNγ and TNFα play an important role during the development of CAC. Studies showed an increased level of these cytokines in AOM/DSS induced mice serum or colon [25,26,27,28].

**Figure 3:**
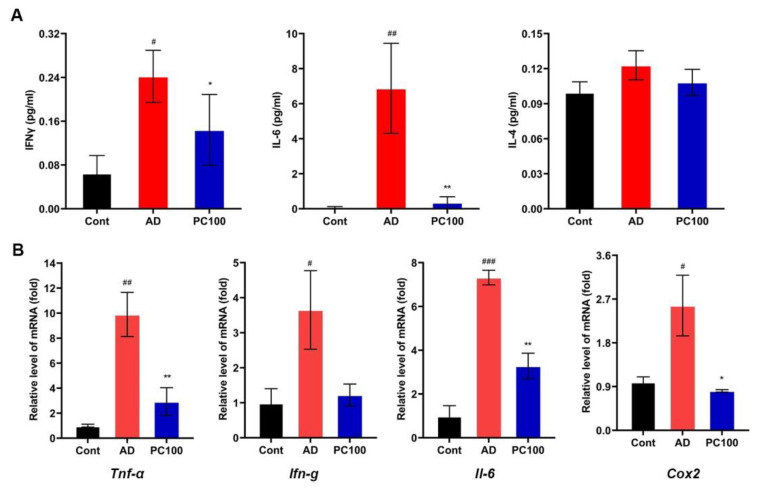
Effect of PC supplementation on proinflammatory mediators and cytokines. (**A**) Concentrations of IFNγ, IL-6 and IL-4 in sera from the Cont, AD and PC100 mice. (**B**) qPCR evaluation of the expression of *Tnf-α*, *Ifn-g*, *Il-6* and *Cox2* relative to that of *Gapdh*. Data were expressed as mean ± SEM (*n* ≥ 5 per group). ^#^ *p* < 0.05; ^##^ *p* < 0.01; ^###^ *p* < 0.001 vs. Cont group; * *p* < 0.05; ** *p* < 0.01 vs. AD group.

We found that PC significantly reduced the levels of IL-6 and IFNγ in mice serum (Figure 3A). Similar results were obtained from the qPCR experiment (Figure 3B). IL-4 is considered as an anti-inflammatory cytokine [29,30] however, PC treatment did not show an obvious effect on the IL-4 level. The expression level of *clooxygenase-2* (*Cox2*), a major proinflammatory enzyme [31], was also inhibited by PC. Collectively, these results demonstrated the anti-inflammatory effect of PC.

### 2.2. PC Treatmend Affected CAC Mice Gut Microbiota

**Figure 4:**
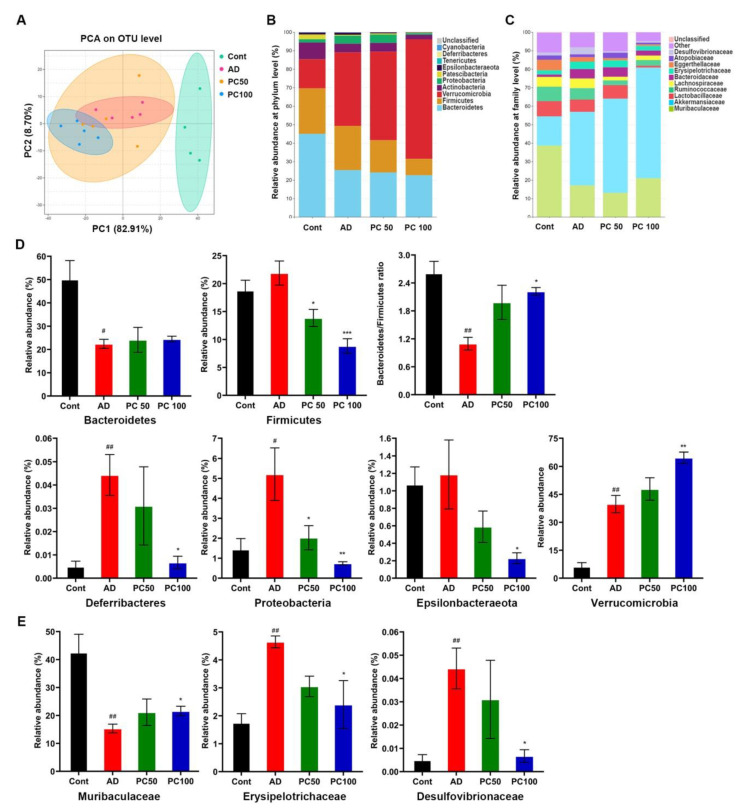
Effects of PC on gut microbiota in AOM/DSS induced CAC mice. PCA analysis on OTU level (**A**) and microbial relative abundance at phylum level (**B**) and family level (**C**) were shown. (**D**) At phylum level, the relative abundance of Bacteroidetes, Firmicutes, Bacteroidetes/Firmicutes ratio, Deferribacteres, Proteobacteria, Epsilonbacteraeota and Verrucomicrobia were affected by PC treatment. (**E**) Comparison of the relative abundance of Muribaculaceae, Erysipelotrichaceae and Desulfovibrionaceae in different groups. Data were expressed as mean ± SEM (*n* ≥ 4 per group). ^#^ *p* < 0.01; ^##^ *p* < 0.01 vs. Cont group; * *p* < 0.05; ** *p* < 0.01; *** *p* < 0.001 vs. AD group.

Numerous research studies have shown the important role of gut microbiota in the development of colorectal cancer. To further investigate whether PC attenuated AOM/DSS induced CAC is related to gut microbiota, we performed 16S rRNA sequence analysis on feces samples collected from mice. The microbial α-diversity indices (Chao, Ace, Shannon and Simpson index) were slightly declined in the AD group without significance. PC treatment significantly decreased the diversity indices (Shannon and Simpson indexes) and the richness indices Ace, indicating that PC can affect the microbial community (Supplementary Figure S1 and Table S1). PCA analysis on OTU level indicated that the microbial community changed among three groups (Figure 4A–C).

We further carried out the relative abundance analysis, at the phylum level, Verrucomicrobia, Bacteroidetes, Firmicutes, Actinobacteria and Proteobacteria were the major phyla in the three groups. Compared to the Cont group, the relative abundance of Firmicutes, Proteobacteria and Deferribacteres were significantly increased in the AD group, while the relative abundance of Bacteroidetes was dramatically decreased (Figure 4D). PC significantly decreased the abundance of Firmicutes, Proteobacteria, Deferribacteres and Epsilonbacteraeota. Interestingly, PC markedly increased the Verrucomicrobia level and the Bacteroidetes/Firmicutes ratio. At the family level, we found a significant decrease in Muribaculaceae and increase in Erysipelotrichaceae and Desulfovibrionaceae in the AD group compared to that of the Cont group, and PC treatment (100 mg/kg) significantly reversed the changes (Figure 4E).

### 2.3. Transcriptome Analysis of Genes Expressed in CAC Mice

**Figure 5:**
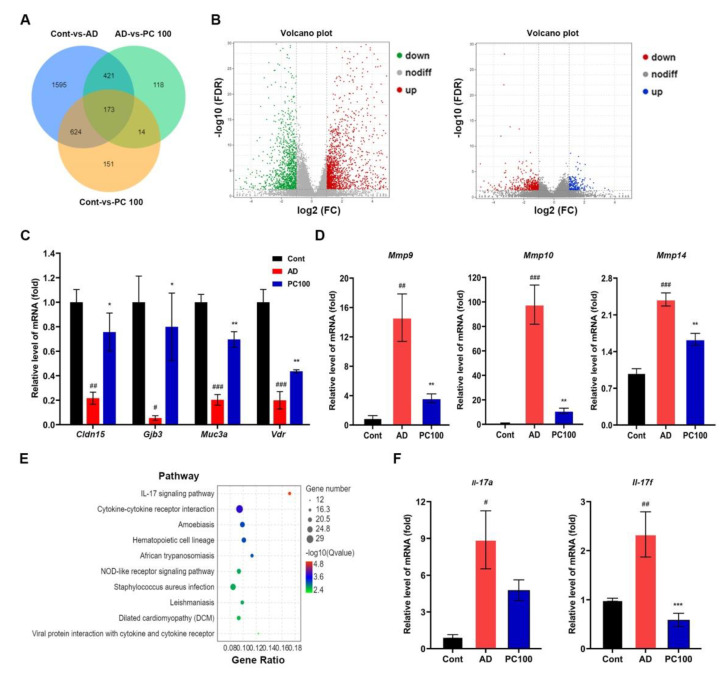
RNA-Seq analysis of DEGs in colon of the Cont, AD and PC100 group mice: (**A**) Venn diagram of DEGs; (**B**) volcanogram of DEGs, left, Cont vs. AD, right, AD vs. PC100; (**C**) Up-regulated DEGs; (**D**) Down-regulated DEGs in PC100 group mice were selected and validated by qPCR analysis; (**E**) KEGG enrichment analysis of DEGs identified from AD vs. PC100; (**F**) qPCR evaluation of the expression of IL-17 pathway members *Il-17a* and *Il-17f* relative to that of *Gapdh*. Three replicates were carried out in the qPCR analysis (*n* = 3). ^#^ *p* < 0.01; ^##^ *p* < 0.01; ^###^ *p* < 0.001 vs. Cont group; * *p* < 0.05; ** *p* < 0.01; *** *p* < 0.001 vs. AD group.

Transcriptome analysis provides an efficient way for the systematic analysis of genes that may be involved in CAC development. We therefore identified the differentially expressed genes (DEGs) between AD mice and PC100 group mice following the cutoff criteria fold-change |log2FC| ≥ 1 and *p*-value < 0.05. A library with size-normalized count for the specimen was generated by making volcano plots for the DEGs as described elsewhere (Figure 5A,B) [32].

Comparison of the AD group and the PC100 group identified 726 DEGs, among which 218 were upregulated and 508 were down-regulated (Supplementary Table S2). These upregulated DEGs included *Muc3a*, *Vdr*, *Cldn15* and *Gjb3*, whereas the downregulated DEGs included *Mmp9*, *Mmp10* and *Mmp14*, our qPCR study further confirmed the results (Figure 5C,D).

Gene ontology (GO) analysis showed that the upregulated and downregulated DEGs were significantly enriched in the cellular process, biological regulation, developmental process etc. (Supplementary Figure S2).

Kyoto Encyclopedia of Genes and Genomes (KEGG) pathway analysis showed that DEGs were mapped to numerous pathways. Figure 5E presented the top ten significantly enriched KEGG pathways of the DEGs (AD vs. PC100). The most significantly enriched pathway was the IL-17 signaling pathway. Others included pathways involved in cytokine-cytokine receptor interaction, such as Amoebiasis, Hematopoietic cell lineage and so on. To verify the KEGG enrichment results, we examined the mRNA expression level of *Il-17a* and *Il-17f* (members of the *Il-17* family). The qPCR results indicated that PC significantly inhibited *Il-17f* expression.

### 2.4. Spearman’s Correlation Analysis of Microbiota and DEGs Regulated by PC

**Figure 6:**
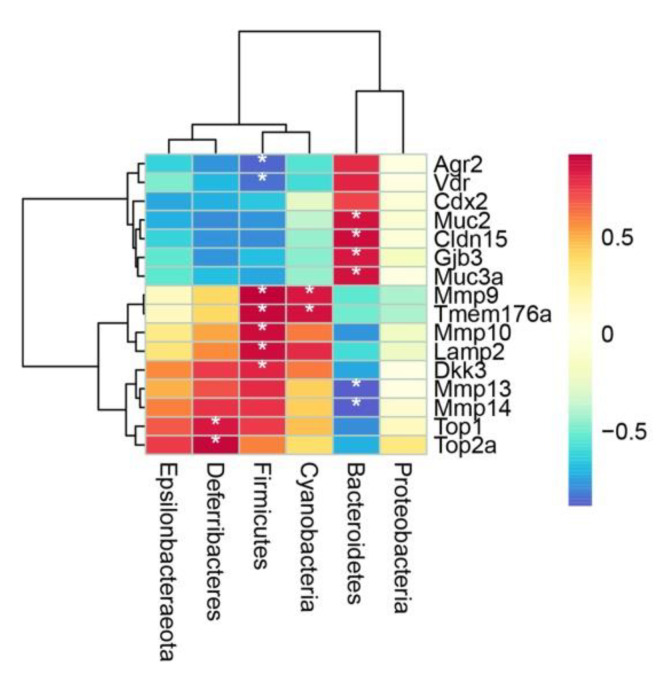
Spearman’s correlation analysis of microbiota and representative DEGs. Red color denotes positive correlation, blue color denotes negative correlation. * *p* < 0.05.

To further investigate the correlation between changes in gut microbiota and the expression level of DEGs, a Spearman correlation analysis between the six phyla and the selected DEGs among three groups was carried out (Figure 6, Supplementary Table S3). Bacteroides was positively correlated with *Muc2*, *Cldn15*, *Gjb3* and *Muc3a*, however, it was negatively correlated with *Mmp13*, *Mmp14* (*p* < 0.05). Firmicutes showed negative correlation with *Agr2* and *Vdr*, and showed positive correlation with *Mmp9*, *Tmem176a*, *Mmp10*, *Lamp2* and *Dkk3* (*p* < 0.05). Deferribacteres was positively correlated with *Top1*, *Top2a* (*p* < 0.05) and Cyanobacteria was positively correlated *Mmp1*, *Tmem176a* (*p* < 0.05).

## 3. Discussion

CRC is one of the most common causes of cancer-related death, epidemiological reports have shown that individuals with IBD are more likely to develop colorectal cancer (CRC) than the general population [33].

CAC progression is influenced by intestinal bacteria in the mucosal layer. Many bacteria, especially Bacteroides, *Escherichia coli* and *Fusobacterium nucleatum*, are involved in CAC progression [6,34].

PC is a blue pigment and has multiple biological functions. It has been utilized in foods and cosmetics, biotechnology, diagnostics and medicines [10]. In vitro studies have shown the antineoplastic values of PC in different cancer cells, such as pancreatic cancer [16], lung cancer [35]. PC can inhibit cell cycle progression, induce cell apoptosis and decrease proinflammatory cytokines through regulating PI3K-Akt, Jak3-Stat3, NF-κb and Wnt signaling pathway [18,19,21,36]. The role of PC in modulation of the gut microbiota in CAC mice has not yet been investigated.

PC intervention increased the bacterial abundance and diversity, and reduced intestinal permeability and increased the intestinal barrier function [22], suggesting a protective role of PC in the gut.

The AOM/DSS model is a chemically induced CAC mouse model employing AOM and DSS carcinogens. When compared with the DMH induced model, the AOM/DSS-induced mouse model has higher reproducibility, mimics a form of inflammatory colorectal cancer in humans and is widely used in CAC studies [37].

In the present study, we demonstrated that orally administered PC markedly inhibited AOM/DSS induced CAC in mice, as evidenced by the tumor number and histopathological examination. PC also inhibited colorectal tumor proliferation, and alleviated colonic inflammation by decreasing the inflammatory cytokines (IFNγ and IL-6) level. As mentioned above, the expression of proinflammatory genes (*Tnf-α*, *Il-6* and *Cox2*) were downregulated, supporting the findings of a previous study, which showed that the application of PC resulted in the decreased protein expression of proinflammatory cytokines IL-1β, IFNγ and TNF-α [19]. While there was a decrease in the genetic expression of *Ifn-γ* in this study, it was not significant.

Nowadays, microbiome and transcriptome have been widely used in biological research to provide a comprehensive understanding of gene expression [32]. As such, 16S rRNA sequence analysis indicated that PC significantly changed the composition of mice gut microbiota by decreasing Firmicutes, Deferribacteres, Proteobacteria, Cyanobacteria and Epsilonbacteraeota at the phylum level. PC treatment increased the Bacteroidetes/Firmicutes ratio.

In an American patient cohort study, a notable increase in Firmicutes was observed when compared to non-adenoma subjects in adenoma biopsies [38]. The appropriate Firmicutes/Bacteroidetes ratio is accepted as a marker in maintaining normal intestinal homeostasis, and a disturbed Firmicutes/Bacteroidetes ratio is regarded as dysbiosis and has been associated with IBD [39]. Proteobacteria is enriched in intestinal pathogens, which can cause inflammation and change intestinal microbiota, and promote the development of IBD [40]. Muribaculaceae is one of the major mucin monosaccharide foragers, a recent study showed that expansion of Muribaculaceae in the gut resulted in the consumption of N-acetylglucosamine and an impeding of the colonization of pathogens such as *Clostridium perfringens*, thereby exerting a protective effect in the gut [41]. The bacterial family Erysipelotrichaceae belongs to the Firmicutes phylum. Studies have revealed the increased abundance of Erysipelotrichaceae in CRC patients and the DMH induced colon cancer animal model [28,42,43], implying that it may be involved in the occurrence and development of CRC. Few research studies have investigated the relationship between Desulfovibrionaceae and CRC, however, many studies reported that it is an endotoxin-producer [44,45,46], endotoxin can promote colorectal cancer cells adhesion and invasion through the regulation of the TLR4/NF-κB pathway [47]. The results of the present study suggest that PC has a positive effect on the gut microbiota, thus exerting a tumor preventive effect.

We also found a dramatic increase in Verrucomicrobia in both the AD and PC treated groups. A recent study showed that natural algal extracts such as ulvan and astaxanthin assist the increase in beneficial microbial populations such as Bacteroidia, Bacilli, Clostridia, and Verrucomicrobia in the gut [48]. We assumed that the increase in Verrucomicrobia might not be an adverse event.

Transcriptomic study identified that DEGs such as *Muc3a* and *Vdr* were up-regulated by PC. Muc3a is a very large cell surface glycoprotein present in columnar epithelial cells of the small intestine and colon [49]. Previous study suggested that Muc3a may inhibit pathogens attached to the intestinal epithelial cells [50]. Studies also revealed that VDR overexpression significantly reduced the sizes and numbers of tumor spheres formed by CRC stem cells [51]. We also found that PC increased the levels of the colon cell junction markers *Cldn15* and *Gjb3*, while it decreased the expression level of *Mmp9*, *Mmp10* and *Mmp14*. This result demonstrated that PC may reduce intestinal permeability and reinforce the intestinal barrier function, thus affecting AOM/DSS induced CAC progression.

KEGG enrichment analysis revealed the IL-17 signaling pathway was one of the most obvious pathways regulated by PC. The qPCR experiment validated the result. However, how PC regulated the IL-17 pathway requires further study.

There are limitations in our research, for example, although our work proposed a potential correlation between specific bacteria and the gene expression profile, we did not show any direct evidence; perhaps fecal transplantation experiments in germ-free mice would be a better way to address the question.

This study investigated the effect of PC on CAC from the perspective of microbiota modulation and can be used as a reference for researchers to further investigate the underlying mechanism of PC in CAC. PC is a promising drug for CAC prevention.

## 4. Materials and Methods

### 4.1. Reagents and Antibodies

PC (extracted from Spirulina platensis, Amax/A280 > 3.5) was purchased from Binmei Biotech (Taizhou, China). PC was dissolved in phosphate buffer solution (PBS) according to the instructions. AOM (cat. no. 25843-45-2) was purchased from Sigma-Aldrich. DSS (cat. no. 160110) was purchased from MP Biomedicals, LLC (Aurora, OH, USA). Primary antibodies for PCNA (13110) were purchased from CST Company (Danvers, MA, USA).

### 4.2. Animal

All animal experiments followed the guidelines for ethical procedures and scientific care given by the Animal Care and Use Committee of the Guangxi University of Chinese Medicine. Wild-type C57BL/6J mice were purchased from Hunan SJA Laboratory Animal Co., Ltd. (Changsha, China). All animals were housed in plastic cages under a 12 h light/dark cycle with free access to water and food. To establish the CAC mice model, the male mice were injected with AOM (10 mg/kg i.p.), exposed to drinking water containing 2% DSS for one week, and then left to drink normal water for two weeks. This treatment was repeated for three cycles.

### 4.3. Histology

At the end of the study, mice were sacrificed by cervical dislocation. The colon was dissected, hematoxylin and eosin (H and E) staining and immunohistochemistry (IHC) analysis was performed. Briefly, the tissues were collected and fixed in 4% paraformaldehyde (PFA) overnight. Then, the tissues were embedded in paraffin wax. The paraffin-embedded tissues were sectioned serially at 5 μm thick. The sections were dewaxed, hydrated, and stained with H and E. For immunohistochemistry, the dewaxed sections were microwaved in the antigen unmasking solution, incubated in 3% hydrogen peroxide, blocked with bovine serum albumin (dissolved in PBS), and incubated with primary antibodies at 4 °C overnight. Finally, the signals were detected by incubating the sections with horseradish peroxidase (HRP)-conjugated secondary antibodies and then 3,3′-diaminobenzidine (DAB) staining colorimetric reagent was used. All images were processed with Adobe Photoshop CS6.

### 4.4. Serum Cytokines Detection

The concentration of IFNγ, IL-6 and IL-4 in serum were determined follow the instructions of the commercial kits (BIORAD, cat NO. 12002798) bought from Shanghai Univ-Biotechnology Co., Ltd. (Shanghai, China). All procedures were performed according to manuals instructions.

### 4.5. RNA Extraction, Gene Microarray and Real-Time PCR

Total RNA was isolated from testes tissues or cells using a TRIzol solution (Invitrogen). For the microarray, the RNA was labeled and hybridized to Affymetrix chips as previously described [52]. One microgram of total RNA from each sample was reverse-transcribed to cDNA in a 20 μL reaction volume. The real-time PCR was performed using a Roche LC480 PCR system, and the mRNA expression level was normalized to *Gapdh* mRNA and analyzed using the comparative cycle threshold method. The primers employed in these experiments are listed in Supplemental Table S4.

### 4.6. Statistical Analysis

All statistical analyses were conducted using SPSS 20.0 analysis software (SPSS Inc., Chicago, IL, USA). Data were analyzed by one-way ANOVA followed by the Tukey’s post hoc test. Bioinformatics analysis of RNA-seq and 16S rRNA sequence, including species abundance, was performed using Omicsmart (Genedenovo Biotechnology Co. Ltd., Guangzhou, China), microbiota data were analyzed by Kruskal–Wallis one-way ANOVA followed by Wilcoxon–Mann–Whitney test (data not normally distributed). Significance was set at *p* < 0.05 for all tests, data were expressed as Mean ± SEM.

## 5. Conclusions

In this study, we demonstrated that PC alleviated inflammation and reduced tumorgenesis in AOM/DSS induced mice. The tumor preventation may be mediated through modulating the gut microbiota and gene expression in colonic cells. PC is a promising drug for clinical prevention and treatment of colorectal cancer.

## Data Availability

All data supporting the conclusions of this article are included in this article.

## Supplementary Materials

The following supporting information can be downloaded at: https://www.mdpi.com/article/10.3390/md20040260/s1. Figure S1. PC decreased the α-diversity of the microbiota in AOM/DSS induced mice model; Figure S2. GO enrichment analysis of differentially expressed genes (DEGs) identified in the study; Table S1. Data of the α-diversity index analysis; Table S2. DEGs were identified in CAC mice with or without PC treatment; Table S3. Data of Spearman’s correlation analysis; Table S4. Primers list of qPCR.
