# Supplementary material for: Phycocyanin Ameliorates Colitis-Associated Colorectal Cancer by Regulating the Gut Microbiota and the IL-17 Signaling Pathway
Source: Mar Drugs. 2022 Apr 9;20(4):260. doi: 10.3390/md20040260 (PMC9030732; doi:10.3390/md20040260)
Supplement: Supplementary file 1 [file marinedrugs-20-00260-s001.zip › marinedrugs-1660453-supplementary.pdf]

Supplemental Fiugures

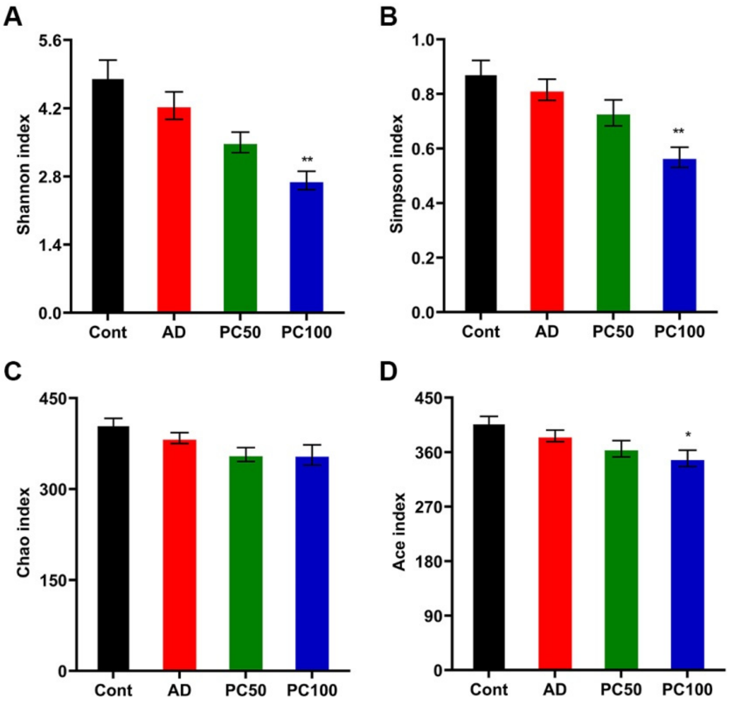

Figure S1. PC decreased the α-diversity of the microbiota in AOM/DSS induced mice model. Bacterial diversity estimated with the Shannon index (A) and Simpson's index (B) and bacterial richness estimated with the ACE value (C) and Chao value (D) were shown. Cont, normal control group; AD, AOM/DSS treated group; PC50, PC low-dose(50mg/kg) group; PC100, PC high-dose(100mg/kg) group;. Data were analyzed by Kruskal-Wallis H non-parametric test and were expressed as mean ± SEM (n = 5 per group).$^{*}p < 0.05$; $^{**}p < 0.01$ vs. AD group..

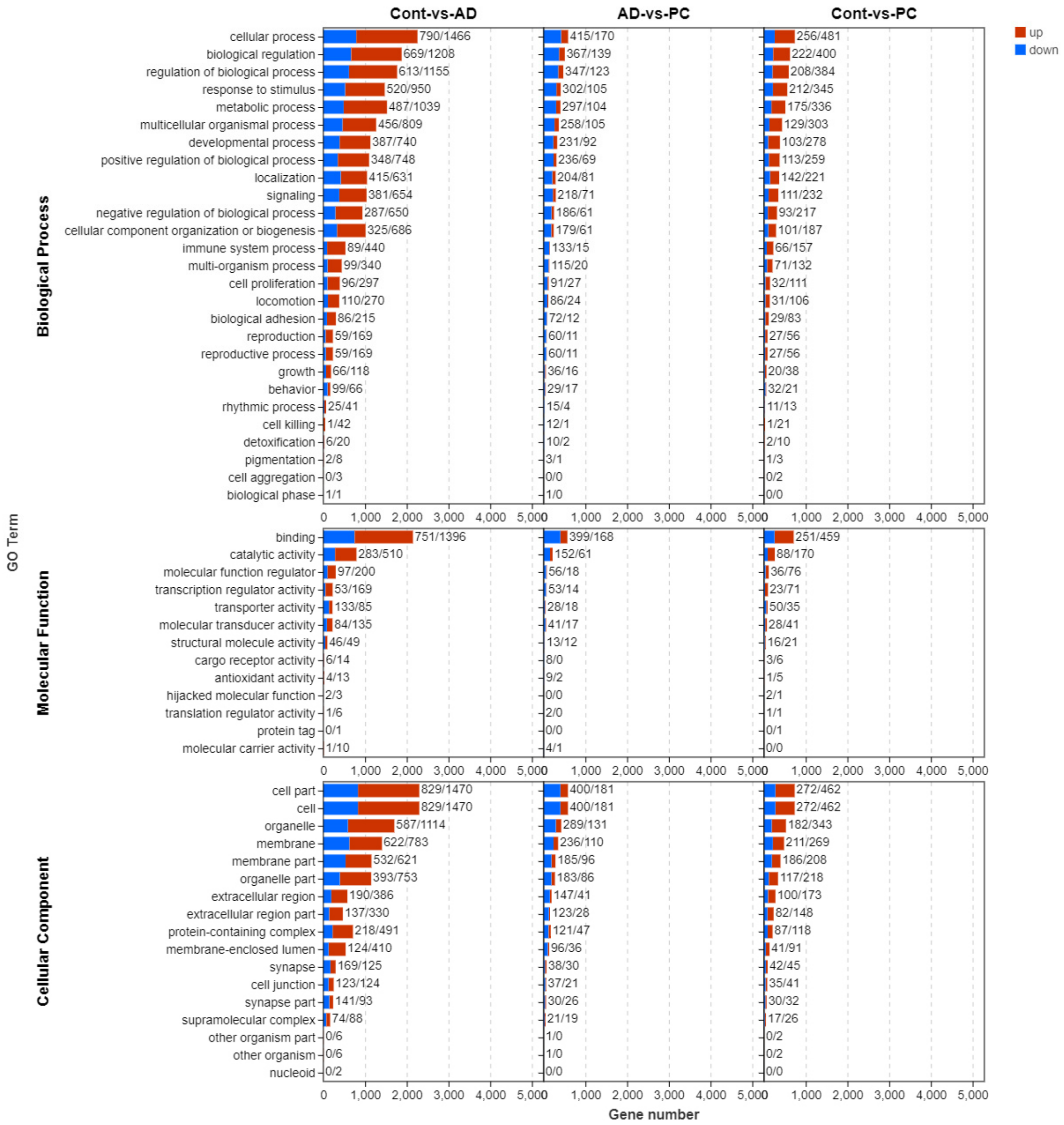

Figure S2. GO enrichment analysis of differentially expressed genes (DEGs) identified in the study. The DEGs were identified following the cutoff criteria fold-change |log2FC| ≥ 1 and P-value < 0.05.
